# A Chemical-Transport-Mechanics Numerical Model for Concrete under Sulfate Attack

**DOI:** 10.3390/ma14247710

**Published:** 2021-12-14

**Authors:** Xuandong Chen, Xin Gu, Xiaozhou Xia, Xing Li, Qing Zhang

**Affiliations:** 1 College of Mechanics and Materials, Hohai University, Nanjing 211100, China; chenxuandong@glut.edu.cn (X.C.); xingu@hhu.edu.cn (X.G.); xiaxiaozhou@163.com (X.X.); hhulixing@hhu.edu.cn (X.L.); 2 College of Civil and Architecture Engineering, Guilin University of Technology, Guilin 541004, China; 3 Guangxi Engineering and Technology Center for Utilization of Industrial Waste Residue in Building Materials, Guilin 541004, China

**Keywords:** external sulfate attack, calcium leaching, aluminate dissolution, mesoscopic concrete, time-dependent boundary condition

## Abstract

Sulfate attack is one of the crucial causes for the structural performance degradation of reinforced concrete infrastructures. Herein, a comprehensive multiphase mesoscopic numerical model is proposed to systematically study the chemical reaction-diffusion-mechanical mechanism of concrete under sulfate attack. Unlike existing models, the leaching of solid-phase calcium and the dissolution of solid-phase aluminate are modeled simultaneously in the developed model by introducing dissolution equilibrium equations. Additionally, a calibrated time-dependent model of sulfate concentration is suggested as the boundary condition. The reliability of the proposed model is verified by the third-party experiments from multiple perspectives. Further investigations reveal that the sulfate attack ability is underestimated if the solid-phase calcium leaching is ignored, and the concrete expansion rate is overestimated if the dissolution of solid-phase aluminate is not modeled in the simulation. More importantly, the sulfate attack ability and the concrete expansion rate is overestimated if the time-dependent boundary of sulfate concentration is not taken into consideration. Besides, the sulfate ion diffusion trajectories validate the promoting effect of interface transition zone on the sulfate ion diffusion. The research of this paper provides a theoretical support for the durability design of concrete under sulfate attack.

## 1. Introduction

Sulfate attack is one of the main factors leading to the performance deterioration of reinforced concrete (RC) structures subjected to sulfate environment [1,2]. The sulfate ion in the external environment diffuses into the concrete and then reacts with the calcium ion in the concrete to form gypsum ($C\bar{S}H_{2}$) [3]. Gypsum further reacts with aluminate in concrete pore solution to form ettringite ($C_{6}A{\bar{S}}_{3}H_{32}$) [3,4]. The expansion of ettringite reduces the pore volume of concrete, thus inhibiting the diffusion of sulfate ions [5,6]. However, the expansion of ettringite also leads to the microcrack [7], which provides the new channels for sulfate ion diffusion and promotes the diffusion of sulfate ion [8,9,10]. It is of great significance to investigate the mechanism of sulfate attack for evaluating the service life of RC structures in a sulfate environment.

The diffusion-reaction process of sulfate ions in concrete has been widely investigated by experiments and numerical simulations [3,11,12] over the past few decades. Xie et al. [11] investigated the diffusion behavior of sulfate ion in concrete by immersion experiments, and the research results showed that the diffusion behavior of sulfate ion in concrete could be described by Fick’s second law. Based on the chemical kinetic reaction equation, Ren et al. [13] investigated the failure mechanism of concrete under sulfate attack by the dry-wet cycle experiment. The dry-wet cycle accelerated the diffusion of sulfate. Furthermore, the experimental results showed that the influence of water transport on sulfate attack of unsaturated concrete should not be ignored [14]. Li et al. [14] established a coupled model of water and sulfate transport in unsaturated concrete by introducing a convection term, and analyzed the effect of the wet-dry cycle on sulfate attack. However, concrete damage and sulfate ion diffusion are studied separately, while the coupling effect between them is ignored. Zuo et al. [15,16] proposed a diffusion-reaction model of sulfate ion in concrete, and investigated the volume strain of concrete caused by sulfate attack. However, the effect of ettringite expansion on sulfate ion diffusion was not considered in Zuo et al.’s model. The expansion of ettringite caused by sulfate attack had a significant effect on sulfate ion diffusion [5,6]. By introducing porosity and crack density functions, Sarkar et al. [9] established a diffusion-reaction-mechanic coupling sulfate attack model. However, this coupling effect is only based on the empirical formula as the coupling factor, and the mechanical behavior of concrete caused by sulfate corrosion is not numerically simulated. Additionally, for the RC structures attacked by chloride and sulfate in coastal areas and salt lake areas, a coupling chloride and sulfate attack model was established to investigate the interaction between sulfate ion and chloride ion [17,18,19].

Although great progress has been made to investigate the diffusion-reaction behavior of sulfate ions in concrete, there still exist many critical ongoing problems. In the actual situation, the sulfate ion intrudes into the concrete and reacts with calcium ion in pore solution to form gypsum. Meanwhile, the dissolution of solid calcium hydroxide (*CH*) and the decalcification of calcium silicate hydrates (*C-S-H*) supplement the calcium ion consumed by the chemical reaction [20]. The leaching of solid-phase calcium increases the porosity of concrete, promotes the diffusion of sulfate ions [21,22], and reduces the strength of concrete [23]. Currently, the leaching effect of solid-phase calcium under sulfate attack was observed by a microscopic test method in the experiments of Li et al. [24]. However, the experimental measure method can only measure the concentration distribution of solid-phase calcium at a certain ingress time, and cannot dynamically show the evolution process of solid-phase calcium with the increase of sulfate ingress time. Additionally, most of the existing sulfate attack models assumed that the concentration of calcium ions in the pore solution was constant and saturated during the process of sulfate attack [3,11,23,25], and ignored the effect of solid-phase calcium leaching and the reduction of solid-phase calcium [26,27], which is inconsistent with the real situation. Therefore, it is necessary to model the dissolution of solid-phase calcium to fully explore the mechanism of sulfate attack. The full coupling of sulfate attack, chemical reaction and mechanical behavior will become a research hotspot in the future.

Additionally, in the process of sulfate attack to concrete, the produced gypsum further reacts with aluminate in concrete pore solution to form ettringite. The aluminate is first dissolved into the pore solution of concrete [23], and then reacts with gypsum in the solution to form ettringite [22,28]. Geng et al. [28] revealed the mechanism of ettringite formation by the reaction between aluminate and gypsum through microscopic experiments. As is known, the aluminates in the concrete include the aluminates in the pore solution and the solid-phase aluminates [29,30,31]. That is to say, the initial concentration of aluminate in pore solution is lower than the total concentration of aluminate in concrete. Besides, only the aluminate in the pore solution reacts with the gypsum generated by the chemical reaction between sulfate ion and calcium ion [32]. With the increase of ingress time, the consumed aluminate in pore solution could be supplemented by the dissolution of solid-phase aluminate until the solid-phase aluminate is completely dissolved. Therefore, in the study of sulfate attack, the initial aluminate concentration in the pore solution should not be regarded as the total aluminate concentration in concrete, and the dissolution of solid-phase aluminate needs to be modeled in the prediction model of concrete deterioration caused by sulfate attack. Unfortunately, most of the existing numerical models of sulfate attack on concrete [9,33,34,35,36,37] assumed that the aluminate in concrete was completely dissolved in the pore solution, ignoring the maximum solubility of the pore solution for aluminate and the existence of solid-phase aluminate.

In this paper, the aim of the present work is to establish comprehensive multiphase mesoscopic numerical model with considering the chemo-transport-mechanical effect for concrete under sulfate attack. The remainder of this manuscript is structured as follows: In Section 2, a meso-numerical model of sulfate attack is proposed to reveal the mechanism of sulfate attack. In Section 3, the numerical simulation of sulfate attack on concrete is realized by numerical method. In Section 4, the reliability of the proposed model is verified by the third-party experiments. In Section 5, a comparative analysis is made to fully reveal the significance of considering the effects of solid-phase calcium leaching, solid-phase aluminate dissolution and time-dependent boundary sulfate ion when simulating external sulfate attack. Our findings reveal previously ignored fundamental aspects of the sulfate attack mechanism, and provide insights for the durability prediction of RC structures.

## 2. Chemo-Transport-Mechanical Model for Concrete under Sulfate Attack

### 2.1. Sulfate Ion Diffusion-Reaction

**Figure 1:**
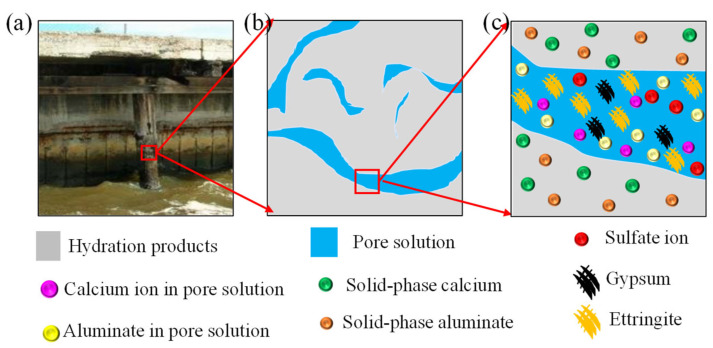
(**a**) Concrete columns in sulfate environment, (**b**) concrete meso-structure, and (**c**) local magnification of concrete meso-structure, respectively.

The sulfate ion in the environment diffuses into the concrete and reacts with the calcium ion in the pore solution of concrete to form $C\bar{S}H_{2}$ [5,17], as illustrated in Figure 1a–c. The chemical reaction can be expressed as Equation (1). In the process of sulfate attack, a part of the sulfate ions is consumed by the chemical reaction, and the remaining sulfate ions continue to diffuse into the concrete driven by the concentration gradient. Therefore, a diffusion-reaction equation could be used to describe the transport process of sulfate ions in the concrete [6,38], which can be expressed as Equation (2).

(1) $$SO_{4}^{2-}+Ca^{2+}+2H\to C\bar{S}H_{2}$$

(2) $$\frac{\partial C_{SO_{4}^{2-}}}{\partial t}=\nabla \cdot \left(D_{SO_{4}^{2-}}\nabla C_{SO_{4}^{2-}}\right)-k_{1}C_{SO_{4}^{2-}}\cdot C_{Ca^{2+}}$$

where *t* is the time variable, $C_{SO_{4}^{2-}}$ is the concentration of sulfate ion with the unit of mol/m^3^, $C_{Ca^{2+}}$ is the concentration of calcium ion (mol/m^3^), $D_{SO_{4}^{2-}}$ is the diffusion coefficient of sulfate ion (m^2^/s), and $k_{1}$ is the chemical reaction rate constant [mol/(m^3^ s)], respectively.

### 2.2. Calcium Ion Reaction-Leaching-Diffusion

**Figure 2:**
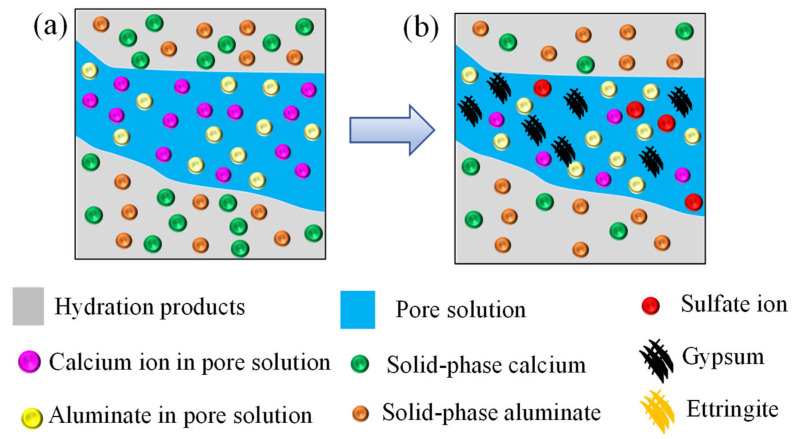
Schematic diagram of calcium reaction-leaching-diffusion process. (**a**) Before sulfate attack, (**b**) after sulfate attack.

Calcium ion in concrete pore solution consumed by the chemical reaction between sulfate ion and calcium ion can be supplemented by the dissolution of calcium hydroxide (CH) and decalcification of hydrated calcium silicate (*C-S-H*) [4,39]. This process is called calcium leaching [4,39]. The leaching of calcium is a complex diffusion and dissolution process, following the law of thermodynamic equilibrium [40]. Before sulfate attack, calcium ion in pore solution is saturated and keeps equilibrium with the solid-phase calcium [41], as shown in Figure 2a. In the process of sulfate attack, a part of the calcium ions in the pore solution is consumed by the chemical reaction between sulfate ion and calcium ion, which leads to the reduction of calcium ion in the pore solution and breaks the solid-liquid calcium ion equilibrium state. Meanwhile, the solid calcium hydroxide begins to leach and replenish the calcium ion in the pore solution to reach a new equilibrium state, as shown in Figure 2b [26]. The leaching rate of solid-phase calcium is related to the concentration of calcium ions in pore solution [42]. The larger the difference between the concentration of calcium ion in the concrete pore solution and the saturated calcium concentration of the concrete pore solution, the faster the leaching rate of solid-phase calcium [43]. Similar to the chemical kinetic reaction equation, the leaching rate of calcium ion can be expressed as

(3) $$\frac{\partial C_{SCa^{2+}}}{\partial t}=-k_{2}\cdot C_{SCa^{2+}}\cdot \left(C_{Ca^{2+},sat}-C_{Ca^{2+}}\right)$$

where $C_{SCa^{2+}}$ is the concentration of solid-phase calcium (mol/m^3^), $k_{2}$ is leaching rate constant of solid-phase calcium [mol/(m^3^ s)], $C_{Ca^{2+},sat}$ is the saturated calcium ion concentration of the concrete pore solution (mol/m^3^), and $C_{Ca^{2+}}$ is the concentration of calcium ion in concrete pore solution (mol/m^3^), respectively.

Moreover, to reflect the consumption of calcium ion in pore solution and the leaching of solid-phase calcium, the chemical reaction kinetic equation of calcium ion in pore solution can be written by Equation (4).

(4) $$\frac{\partial C_{Ca^{2+}}}{\partial t}=\nabla \left(D_{Ca^{2+}}\nabla C_{Ca^{2+}}\right)-k_{1}C_{SO_{4}^{2-}}\cdot C_{Ca^{2+}}-\frac{\partial C_{SCa^{2+}}}{\partial t}$$

where $D_{Ca^{2+}}$ is the diffusion coefficient of calcium ion in pore solution (m^2^/s).

### 2.3. Gypsum and Aluminate Chemical Kinetic Reaction

**Figure 3:**
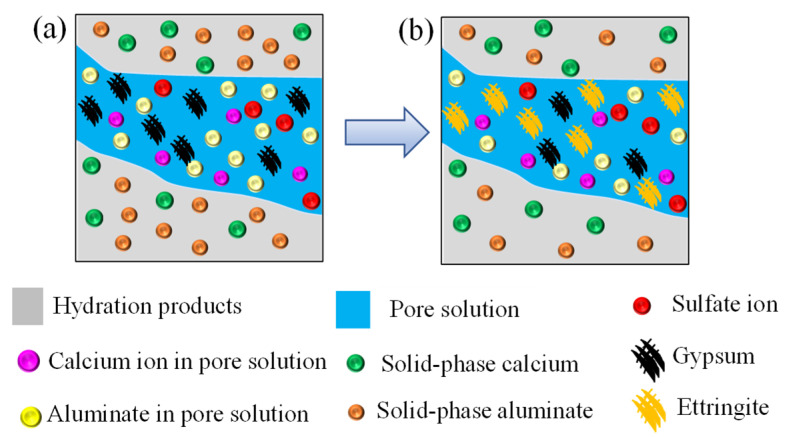
Schematic diagram of chemical reaction process of gypsum and aluminate. (**a**) Before chemical reaction, and (**b**) after chemical reaction.

The gypsum ($C\bar{S}H_{2}$) generated by the chemical reaction between sulfate ion and calcium ion further reacts with the aluminates, i.e., tricalcium aluminum (*C_3_A*), tetra calcium aluminate (*C_4_AH_13_*), and monosulfate ($C_{4}A\bar{S}H_{12}$) in concrete pore solution to form ettringite ($C_{6}A{\bar{S}}_{3}H_{32}$), as shown in Figure 3a,b. This process can be expressed by Equations (5)–(7) [17,44,45,46].

(5) $$3C\bar{S}H_{2}+C_{3}A+26H\to C_{6}A{\bar{S}}_{3}H_{32}$$

(6) $$3C\bar{S}H_{2}+C_{3}AH_{13}+14H\to C_{6}A{\bar{S}}_{3}H_{32}+CH$$

(7) $$2C\bar{S}H_{2}+C_{4}A\bar{S}H_{12}+16H\to C_{6}A{\bar{S}}_{3}H_{32}$$

Similar to the previous works [3,40], to simplify the calculation, the chemical reactions of Equations (5)–(7) could be lumped in a single expression, as follows:

(8) $$q\cdot C\bar{S}H_{2}+CA\to C_{6}A{\bar{S}}_{3}H_{32}$$

where *CA* is equivalent aluminate concentration (mol/m^3^) with $CA=\lambda_{1}C_{3}A+\lambda_{2}C_{4}AH_{13}+\lambda_{3}C_{4}A\bar{S}H_{12}$, *q* is the stoichiometric weighted coefficient with $q=3\lambda_{1}+2\lambda_{2}+3\lambda_{3}$, and $\lambda_{i}$ is the fraction of the component of aluminate, respectively. Therefore, the formation rate of gypsum can be expressed as [43]:

(9) $$\frac{\partial C_{gpy}}{\partial t}=k_{1}\cdot C_{SO_{4}^{2-}}\cdot C_{Ca^{2+}}-k_{2}C_{gpy}\cdot C_{CA}$$

where $C_{gpy}$ is the concentration of gypsum (mol/m^3^).

Since the chemical reaction process occurs in the concrete pore solution, the chemical reaction between gypsum and aluminate can also be described by a reaction-diffusion-dissolution process, written as Equation (10) [20,47,48]:

(10) $$\frac{\partial C_{CA}}{\partial t}=\nabla \left(D_{CA}\nabla C_{CA}\right)-k_{3}C_{gpy}\cdot C_{CA}-\frac{\partial C_{SCA}}{\partial t}$$

where $C_{CA}$ is the concentration of aluminate in pore solution (mol/m^3^), $D_{CA}$ is diffusion coefficient of aluminate (m^2^/s), $C_{SCA}$ is the concentration of solid-phase aluminate (mol/m^3^), and $k_{3}$ is the chemical reaction rate constant [mol/(m^3^ s)], respectively. 

Similar to the leaching process of solid-phase calcium, the dissolution process of solid-phase aluminate can be described by Equation (11) [32,35]:

(11) $$\frac{\partial C_{SCA}}{\partial t}=-k_{4}\cdot C_{SCA}\cdot \left(C_{CA,sat}-C_{CA}\right)$$

where $k_{4}$ is the dissolution rate constant of solid-phase aluminate [mol/(m^3^ s)], and $C_{CA,sat}$ is the saturated aluminate concentration in the pore solution of concrete (mol/m^3^).

### 2.4. Chemical Damage

The ettringite produced by sulfate attack has expansibility, which will produce expansion pressure on the concrete pore wall. Once the expansion pressure exceeds the limit strength of concrete, microcracks appear and the mechanical performance of concrete deteriorates [34,47,49]. Two kinds of expansion pressure theories are widely accepted to understand the mechanism of expansion pressure. One is ettringite volume expansion theory [38,50] and the other is crystallization pressure theory [51,52]. In this work, the ettringite volume expansion theory is adopted to analyze the damage of concrete. According to the continuum mechanics, the crack density of $C_{d}$ is introduced to quantitatively describe the degradation of concrete mechanical performance caused by sulfate attack [9,16], which can be described as follows:

(12) $$C_{d}=k{\left(1-\frac{\varepsilon_{th}}{\varepsilon }\right)}^{m}$$

where $C_{d}$ is the crack density, *k* and *m* are the empirical parameters, $\varepsilon_{th}$ is the threshold strain at which the microcracks start forming, and ε is the volume expansive strain caused by the ettringite, respectively. Moreover, the volume expansive strain can be defined as [9,10,15]:

(13) $$\varepsilon =\max\left(\frac{1}{3}\left(\frac{\Delta V}{V}-f\varphi_{0}\right),0\right)$$

where $\frac{\Delta V}{V}$ is the total pore volumetric change rate due to the formation of ettringite and the calcium leaching, $\varphi_{0}$ is the initial porosity of concrete, f is the volume fraction of the initial porosity being filled before the expansion. f is within the range of 0~1 [53].

It is assumed that the expansion caused by the formation of gypsum is negligible [54]. The change of pore volume in concrete is mainly caused by the leaching of solid-phase calcium and the ettringite expansion. The leaching of solid-phase calcium leads to the formation of new pores, resulting in the increase of pore volume in concrete [34,55]. While the expansion of ettringite leads to the decrease of the pore volume of concrete. The total pore volumetric change rate in concrete is calculated by Equation (14) [54,56]:

(14) $$\frac{\Delta V}{V}=\left(C_{CA_{0}}-C_{CA}\right)\cdot v_{CA}-\left(C_{SCa0}^{}-C_{SCa}^{}\right)\cdot v_{CH}$$

where $C_{SCa0}^{}$ is the initial solid-phase calcium concentration (m^3^/mol), $C_{CA_{0}}$ is the initial aluminate concentration (mol/m^3^), $v_{CH}$ is the unit volume change rate of concrete pore caused by leaching of solid-phase calcium (m^3^/mol), and $v_{CA}$ is the unit volume change rate of concrete pore caused by consumption of aluminate (m^3^/mol), respectively. The detailed calculation process can refer to the literature [35].

The expansion rate of concrete can be calculated by the linear strain obtained at each point, which can be written by Equation (23) [22,44].

(15) $$\varepsilon_{l}\left(t\right)=\frac{\Delta l\left(t\right)}{L}=\frac{\int_{0}^{L}\varepsilon \cdot \Delta d\cdot dy}{L}$$

where $\varepsilon_{l}\left(t\right)$ is the expansion rate of concrete, Δl(t) is the expansion length of the specimen, L is the initial length of the specimen, and Δd is the length of the element, respectively. 

### 2.5. Effective Diffusion Coefficient of Sulfate Ion

In the process of sulfate attack, the gypsum further reacts with the aluminate to form ettringite. On the one hand, the formation of ettringite makes the porosity of concrete decreasing and the diffusion channel of sulfate ion narrow, and thus inhibits the diffusion of sulfate ion [9]. On the other hand, once the expansion pressure produced by ettringite reaches the limit strength of concrete, microcracks will appear in concrete [50,53], which provides new channels for the diffusion of sulfate ion and accelerates the diffusion of sulfate ion.

The effects of the porosity and microcrack on the diffusivity of sulfate ion are considered simultaneously in the calculation of the effective diffusion coefficient of sulfate ion. Based on the migration and porosity test results obtained by Zhang et al. [57], the change of diffusivity caused by the change of porosity can be calculated by using an empirical equation [5]:

(16) $$H_{D}\left(\varphi \right)=\frac{\exp\left(\varphi /v_{c}\right)}{\exp\left(\varphi_{0}/v_{c}\right)}$$

where $H_{D}\left(\varphi \right)$ is the adjustment coefficient of sulfate ion diffusion coefficient dependent on the porosity change, $v_{c}$ is the volume fraction of cement, and $\varphi_{0}$ is the initial porosity, respectively. The initial porosity could be calculated according to the classical powers’ model [58]:

(17) $$\varphi_{0}=v_{c}\cdot \left(\frac{w/c-0.17\alpha }{w/c+0.32}\right)$$

where α is the hydration degree of concrete ranging from 0 to 1, and *w*/*c* is a water-cement ratio. When *w*/*c* is less than 0.5, α can be expressed as [50,59]:

(18) α=1−exp(−3.15×w/c)

The porosity of concrete after sulfate attack is the difference between the initial porosity and the porosity reduced by chemical reaction, which can be expressed as Equation (19).

(19) $$\varphi =\max\left(\varphi_{0}-\frac{\Delta V}{V},\;\;\;\;0\right)$$

When the crack density is sparse, the mean-field can be used to study the effect of crack on sulfate ion diffusion. The diffusion coefficient can be modified by the crack density [8]. However, when macro cracks appear, the effect of seepage on sulfate ion diffusion needs to be considered [9]. Therefore, the effect of ettringite expansion pressure on the sulfate diffusivity can be expressed by piecewise functions, as follows [26,40]:

(20) $$H_{D}\left(C_{d}\right)=\left\{ \begin{array}{l} \left(1+\frac{32}{9}C_{d}\right)\;\;\;\;\;\;\;\;\;\;\;\;\;\;\;\;\;\;\;\;\;\;\;\;\;\;\;\;\;\;\;\;\;\;C_{d}\leq C_{dc}\;\;\;\;\;\;\;\;\;\;\;\;\;\\ \left(1+\frac{32}{9}C_{d}\right)+\frac{{\left(C_{d}-C_{dc}\right)}^{2.3}}{C_{de}-C_{d}}\;\;\;\;\;\;\;\;\;\;\;\;\;\;C_{dc}<C_{d}<C_{d}{}_{e} \end{array}\right.$$

where $C_{dc}$ is the conduction percolation threshold, and it is determined to be 0.182 [60]. Below the conduction percolation threshold, the crack density is sparse. $C_{d}{}_{e}$ is the rigidity percolation threshold at which the cluster of cracks transects the volume, and it is determined to be 0.712 [61].

### 2.6. Boundary Sulfate Ion Concentration

**Figure 4:**
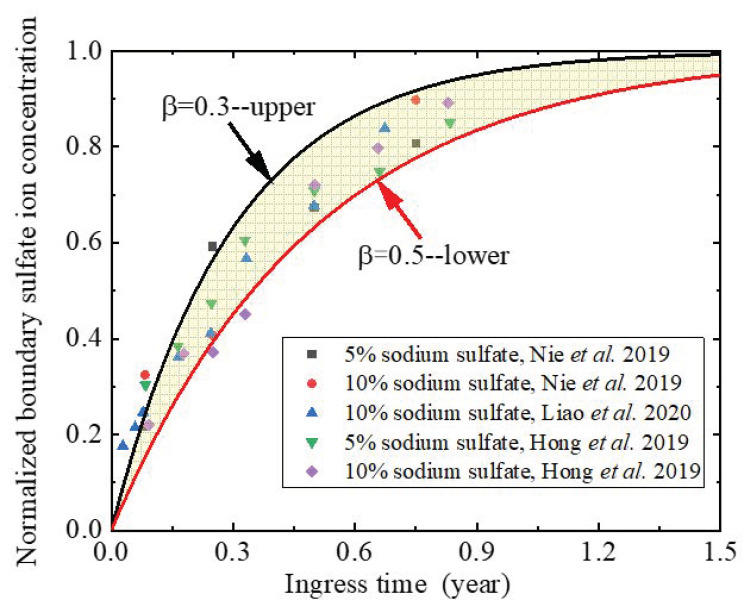
Profiles of boundary sulfate ion concentration.

Many boundary sulfate ion concentrations are collected from experiments [9,33,57] to analyze the characteristics of sulfate ion concentration on the ingress surface. Although these experimental sets [9,33,57] are different at the aspects of water-binder ratio, mineral admixtures, and concentration of sulfate ion solution, the change trends of the boundary sulfate ion concentrations with ingress time are similar, as shown in Figure 4. It is apparent from Figure 4 that with the increase of sulfate ingress time, the boundary sulfate ion concentration increases sharply at the initial stage of sulfate attack, and then tends to be stable. A similar phenomenon has also been reported when studying the chloride attack on concrete [54,57]. This indicates that the concentration of boundary sulfate ion is time-dependent rather than a constant [9,10]. 

Through regression analysis, the boundary sulfate ion concentration can be expressed by an exponential function, as follows:

(21) $$C_{s}=C_{s,\max}\left(1-\exp\left(\frac{t}{\beta }\right)\right)$$

where $C_{s}$ is the boundary sulfate ion concentration (mol/m^3^), $C_{s,\max}$ is the maximum boundary sulfate ion concentration (mol/m^3^), and β is the shape factor and can be obtained from the experimental data, respectively. Figure 4 displays that most of the experimental data fall into the zone between two boundary sulfate ion concentration cures with shape coefficients of 0.3 and 0.5, respectively. This implies that the proposed time-dependent function of boundary sulfate ion concentration can well describe the time-dependent characteristics of boundary sulfate ion concentration.

## 3. Numerical Simulation

**Figure 5:**
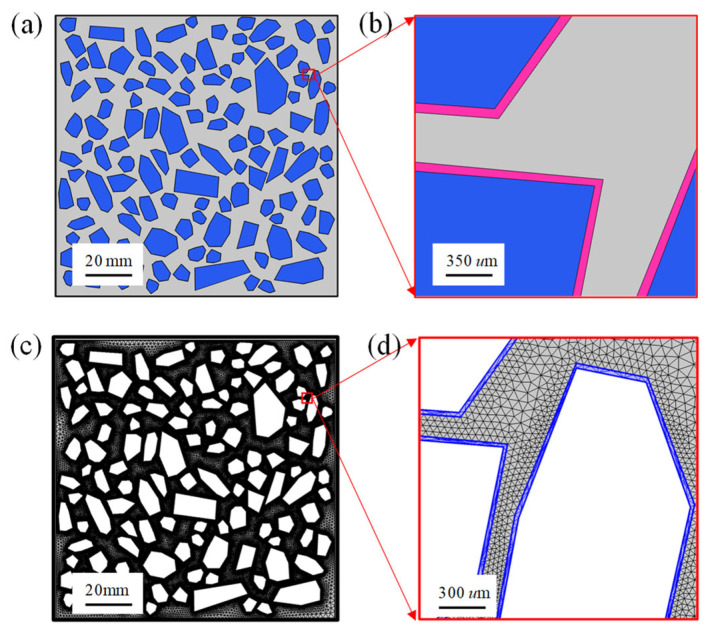
Schematic of 2D multi-phase meso structure of concrete. (**a**) Meso-structure of concrete, (**b**) local magnification of the meso-structure, (**c**) finite element mesh, and (**d**) local magnification of the finite element mesh, respectively.

In this paper, concrete is regarded as a three-phase composite material composed of aggregate, mortar, and interface transition zone (ITZ) [62,63], as shown in Figure 5a,b. thickness of ITZ is related to many factors such as construction technology, water-cement ratio, curing conditions, and mineral admixtures [64]. Generally, the thickness of ITZ ranges from 20 µm to 100 µm [52,64,65]. In this work, the thickness of the ITZ obeys normal distribution, and the average thickness of ITZ is 60 µm and the variance is 10 µm. Moreover, the geometric section size of concrete used in this paper is 100 mm × 100 mm. The aggregate ratio of concrete is 45%, the maximum particle size of aggregate is 20 mm and the minimum particle size is 5 mm. The minimum spacing between two aggregates is 0.1 mm. It is worth noting that the actual three-dimensional (3D) concrete aggregate grading curve needs to be transformed into a two-dimensional (2D) concrete aggregate grading curve in the process of concrete aggregate delivery, as expressed by Equation (22). The random generation algorithm of concrete multiphase meso structure is similar to our previous work [59,64].

(22) $$p\left(d\right)=1.065{\left(\frac{d}{d_{m}}\right)}^{0.5}-0.053{\left(\frac{d}{d_{m}}\right)}^{4}-0.012{\left(\frac{d}{d_{m}}\right)}^{6}-0.0045{\left(\frac{d}{d_{m}}\right)}^{8}-0.0025{\left(\frac{d}{d_{m}}\right)}^{10}$$

where d is the diameter of aggregate (mm), $d_{m}$ is the maximum diameter of aggregates (mm), and P is the cumulative percentage passing a sieve with aperture diameter d, respectively.

Moreover, due to the wall effect of aggregate and the insufficient hydration of cement [66], the porosity of ITZ is 2~3 times of that of the mortar matrix [67]. Moreover, the connectivity of pores in ITZ is better than that in mortar matrix [68,69]. Therefore, the diffusion coefficient of sulfate ion in ITZ is larger than that in mortar matrix. Based on experimental data, the relationship among the ITZ thickness, the diffusion coefficient in ITZ and the diffusion coefficient in mortar matrix established by Zhao et al. [70] can be adopted.

(23) $$\frac{D_{ITZ}}{D_{m}}=\frac{139.434}{u_{ITZ}}+1.0$$

where $D_{ITZ}$ is the diffusion coefficient of sulfate in the ITZ (m^2^/s), $D_{m}$ is the diffusion coefficient of sulfate in the cement mortar zone (m^2^/s) and $u_{ITZ}$ is the thickness of ITZ (µm), respectively.

**Figure 6:**
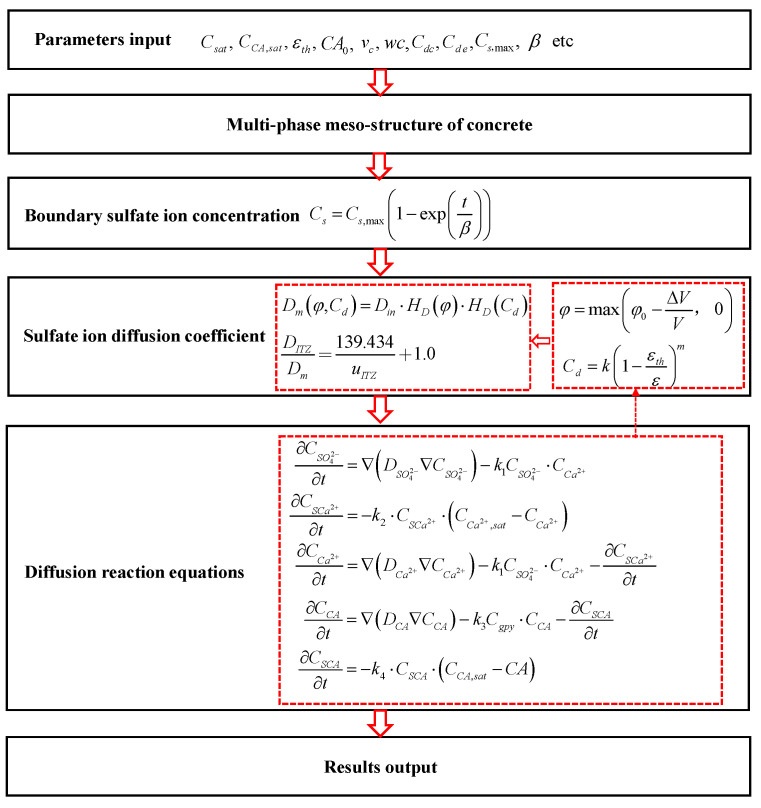
Flow chart of the derivation of the proposed multiphase numerical model.

Additionally, compared with ITZ and mortar, the diffusion coefficient of sulfate ion in aggregate is two orders of magnitude smaller [70,71]. Therefore, the aggregate is generally regarded as an impermeable body [59,64,72], and thus the sulfate ion diffusion in aggregates can be ignored in the numerical simulation. Moreover, the finite element mesh in the simulation is shown in Figure 5c,d, and there are more than 10 million degrees of freedom. In addition, to fully reveal the mechanism of sulfate attack, the leaching of solid-phase calcium and the dissolution of aluminate, and the time-dependent characteristics of boundary sulfate ion concentration are considered simultaneously in the proposed sulfate attack model. The flow chart of the derivation of the proposed multiphase numerical model is presented in Figure 6, and the main parameters used in the numerical simulation are listed in Table 1.

## 4. Model Validation

### 4.1. Distribution of Sulfate Ion Concentration

To verify the reliability of the proposed model, the sulfate attack experimental results by Xie et al. [11] are compared with the present numerical simulation results. In the experiments [11], the water-binder ratio of concrete was 0.485, the diameter of concrete specimens was 100 mm, and the length was 200 mm, respectively. The top and bottom surfaces of the concrete specimens were covered with epoxy resin for sealing, and then soaked in 5% sodium sulfate solution. The ultra-violet and visible spectrophotometer (UVPC) [74] was used to measure the sulfate ion concentration from the powder collected at depths of 5.5 mm, 9.5 mm, 13.5 mm, and 17.5 mm, respectively. In the simulation, the maximum sulfate ion concentration on the surface ($C_{s,\max}$) is 110 mol/m^3^, which is the same as that in Reference [11]. The initial concentrations of hydrated calcium silicate (*C-S-H*) and calcium hydroxide (*CH*) are calculated according to the method of Wan et al. [27]. The parameters used in the simulation are shown in Table 2.

**Figure 7:**
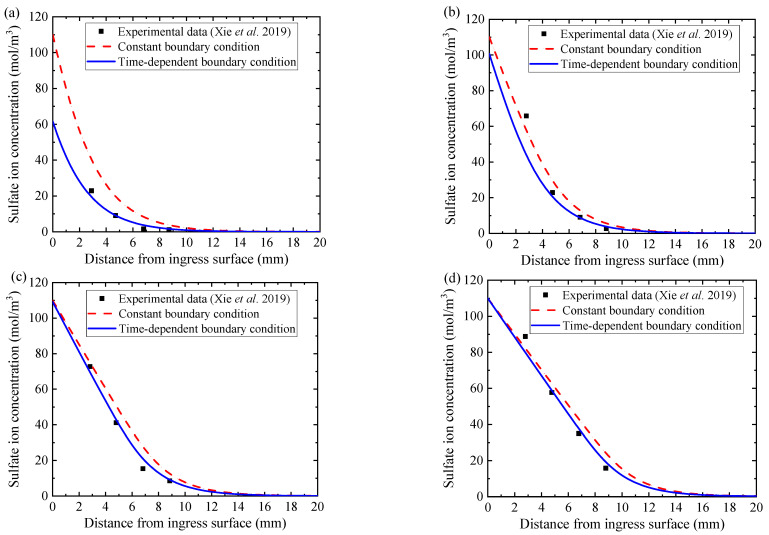
Sulfate ion concentration distribution profiles of the experimental results of Xie et al. [13] and simulation results on different ingress time: (**a**) 30th day, (**b**) 90th day, (**c**) 180th day, and (**d**) 270th day, respectively.

Furthermore, the sulfate attack model either with constant boundary sulfate ion concentration or with time-dependent boundary sulfate ion concentration is calculated for comparison. The simulation results are plotted by the red dotted curves and the blue solid curves in Figure 7a–d, together with the experimental results [11] plotted with black square scatters. The experimental results display that with the increase of sulfate ion ingress time, both the ingress depth and the concentration of sulfate ion gradually increase. Moreover, at the early stage of sulfate attack as the red dotted curve in Figure 7a, the numerical simulation results of the case with a constant boundary condition are significantly higher than the experimental results. With the increase of ingress time, its numerical simulation results are still higher than the experimental data, as shown by the red dotted curves in Figure 7b,c. However, the numerical simulation results of the proposed model with time-dependent boundary sulfate ion concentration are always close to the experimental data in the whole process of sulfate attack, as shown in Figure 7a–d by blue solid curves. Therefore, it is evident that the proposed model with time-dependent boundary sulfate ion concentration is reasonable, reliable, and more consistent with the actual situation.

### 4.2. Expansion Rate of Concrete 

Furthermore, the reliability of the concrete expansion rate calculated by the numerical simulation can be verified by the experimental results of Rozière et al. [75]. Rozière et al. [75] experimentally investigated the performance deterioration process of mortar specimens under sulfate attack. The hexahedron specimens with dimensions of 20 mm × 20 mm × 160 mm were cast using Portland cement CEM 52.5R, and the water-binder ratio of concrete was 0.5. They were immersed in a 3% sodium sulfate solution at 23 °C for more than 400 days. The relevant parameters in the numerical simulations are shown in Table 3. In addition, the numerical simulation results of Qin et al. [40] are extracted to be a comparison.

**Figure 8:**
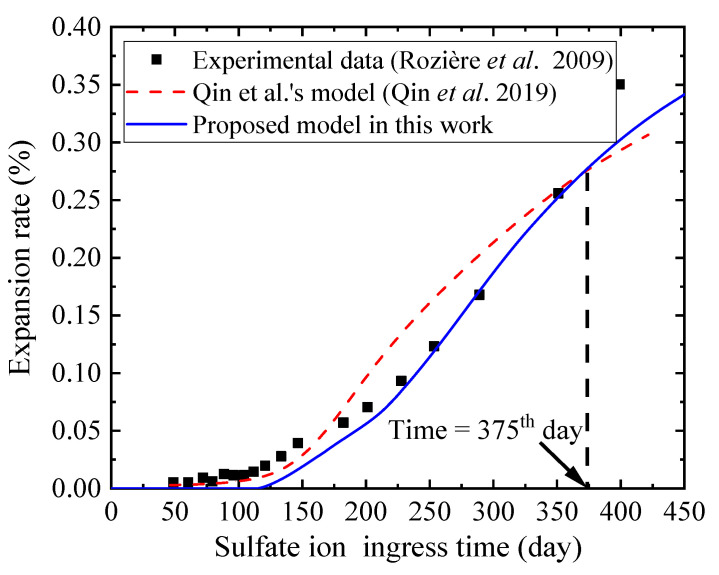
Concrete expansion rates measured by experiments and numerical simulations.

The concrete expansion rate of specimen calculated by ourselves, by Qin et al., and measured by Rozière et al. are shown in Figure 8. It is obvious from Figure 8 that in the early stage of sulfate attack, for which the ingress time is lower than 375 days, the concrete expansion rate calculated by Qin*’*s model is higher than that of our model and the experiment results. While in the later stage of sulfate attack, for which the ingress time is higher than 375 days, the concrete expansion rate calculated by Qin’s model is lower than that of our model and the experiment results. This can be attributed to the initial concentration of aluminate in pore solution being the total concentration of aluminate in concrete in Qin’s model, without considering the dissolution of solid-phase aluminate. Specifically, according to Equation (10), the high initial aluminate concentration leads to a high formation rate of ettringite in the early stage of sulfate attack, resulting in the calculated concrete expansion rate higher than the experimental concrete expansion rate. Moreover, with the increase of ingress time, the concentration of aluminate decreases gradually, and the consumption of aluminate cannot be replenished, correspondingly resulting in a decrease of ettringite formation rate. Moreover, the correlation coefficient between numerical simulation and experimental data is 0.98, which indicates that the numerical simulation results are in good agreement with the experimental results.

## 5. Result Analysis and Discussion 

### 5.1. Sulfate Ion Diffusion

**Figure 9:**
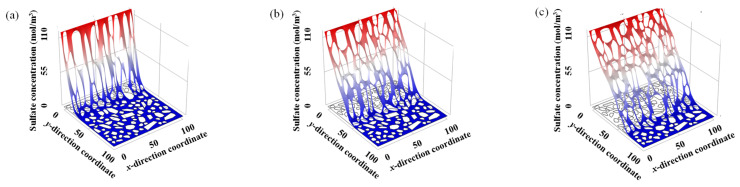
Spatial distribution of sulfate ion concentration at three different ingress time. (**a**) 1st year, (**b**) 5th year, and (**c**) 10th year, respectively.

The spatial distribution of sulfate ion concentration of concrete samples with *w/c* = 0.5 and aggregate volume fraction of 62.4% at three different ingress times (i.e., 1st year, 5th year, and 10th year) is calculated, as shown in Figure 9. It clearly illustrates that both the ingress depth and the concentration of sulfate ion gradually increase with the increase of sulfate ion ingress time, as depicted in Figure 9a–c. For example, the maximum ingress depth is 10.3 mm after 1 year of sulfate ion ingress, while it is 45.7 mm after 5 years of sulfate ion ingress.

**Figure 10:**
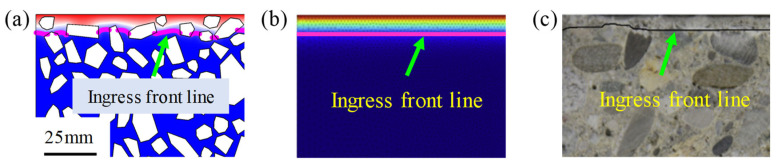
Ingress front lines of sulfate ion concentration from (**a**) present numerical simulation, (**b**) numerical simulation in literature [36], and (**c**) experiments in literature [36], respectively.

Furthermore, due to the different sulfate ion diffusion characteristics of different components in concrete, the random aggregates in concrete leads to the non-uniformity distribution of sulfate ion concentration at the same ingress depth. Therefore, the ingress front line of sulfate ion concentration of the multi-phase mesoscopic model is curvilinear and discontinuous, as shown in Figure 10a. This is quite different from existing models [18,46,48], which regarded concrete as an ideal homogeneous material. In their models, the distribution of sulfate ion concentration is uniform at the same ingress depth and the ingress front line is straight, as shown in Figure 10b. Moreover, the ingress front line of sulfate ion concentration is also curvilinear rather than straight in experiments [36], as shown in Figure 10c. Therefore, the proposed model with a random distribution of aggregates is more consistent with the actual situation.

**Figure 11:**
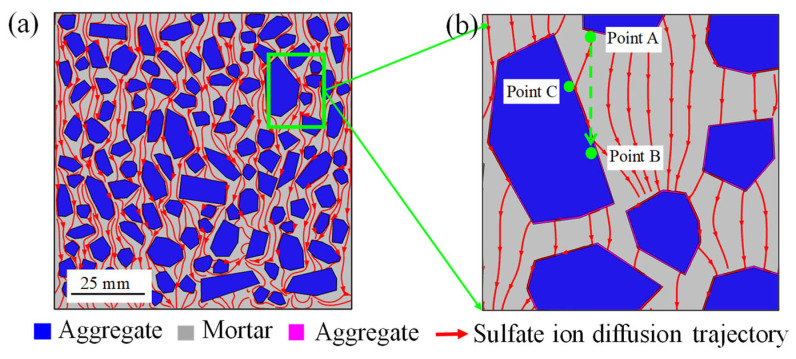
Sulfate ion diffusion trajectories in meso-structures of concrete. (**a**) Overall concrete section, and (**b**) local magnification.

To further reveal the diffusion mechanism of sulfate ion in the multiphase mesostructure of concrete, the diffusion trajectories of sulfate ions is demonstrated in Figure 11a,b. The aggregate lengthens the diffusion paths of sulfate ions, indicating that aggregate has a hindering effect on the diffusion of sulfate ions [64]. Locally enlarging Figure 11a, it is surprising to find that the sulfate ion in the mortar zone tends to diffuse into ITZ first, rather than directly diffuses in the mortar zone, as shown in Figure 11b. For example, sulfate ion at point A does not diffuse to point B along a straight line. Instead, the sulfate ion at point A diffuses first to the ITZ and then along ITZ to point B. This demonstrates that ITZ is a fast channel for sulfate ion diffusion. This phenomenon has not been reported in previous studies regarding concrete as a single-phase homogeneous material. Consequently, the mechanism of ITZ promoting sulfate diffusion in concrete can be well understood through the mesoscale multiphase modeling.

### 5.2. Influence of Solid-Phase Calcium Leaching

**Figure 12:**
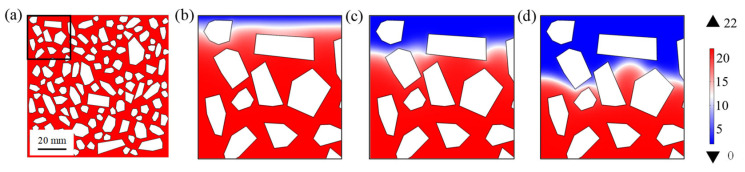
Distributions of calcium ion concentration in concrete pore solution on different ingress time. (**a**) Before sulfate attack, (**b**) 60th day, (**c**) 180th day, and (**d**) 360th day of sulfate attack, respectively.

The distributions of calcium ion concentration in concrete pore solution, considering the leaching of solid-phase calcium, are shown in Figure 12. It should be mentioned that only the area within the black outline in Figure 12a is shown in Figure 12b–d. With the increase of sulfate ingress time, the calcium ion in concrete pore solution near the ingress surface is completely consumed, and the sink term in the chemical kinetic reaction Equation (1) for this region would be equal to 0, which greatly promotes the diffusion of sulfate ion.

**Figure 13:**
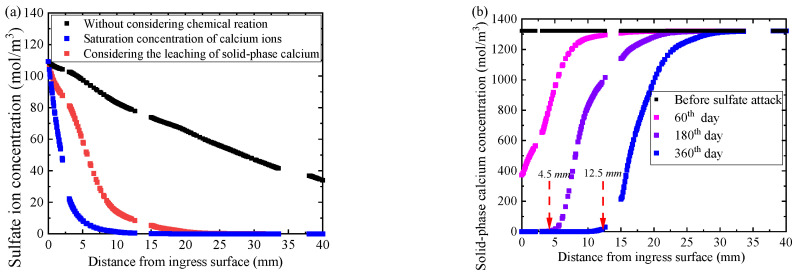
(**a**) Concentration distributions of sulfate ion in the concrete pore solution, and (**b**) concentration distribution of solid-phase calcium ion in the concrete on the 180th day of sulfate attack.

For comparison, the concentration distribution of sulfate ion in concrete without considering the chemical reaction between sulfate ion and calcium ion in pore solution is shown by the black square scatters in Figure 13a. In the process of sulfate attack, a part of sulfate ions is consumed by the chemical reaction, and the remaining sulfate ions continue to diffuse into the concrete. According to the diffusion-reaction equation of sulfate ion (i.e., Equation (1)), the higher the concentration of calcium ion in the pore solution, the more the sulfate consumption, and the less the remaining sulfate ion in the pore solution. The red square scatters and the blue square scatters in Figure 13a represent the concentration distribution of the remaining sulfate ion in concrete with and without considering the leaching of solid-phase calcium. It also decreases gradually due to the continuous decrease of solid-phase calcium, and thus the consumption of sulfate ions is reduced, remaining a relatively large number of sulfate ions in the pore solution. Therefore, by considering the calcium leaching, the sulfate ion concentration in the concrete is higher and the ingress depth is deeper than the results without considering the calcium leaching.

In addition, before sulfate attack, the solid-phase calcium in the concrete is uniformly distributed (herein, 1300 mol/m^3^), as shown by the black square scatters in Figure 13b. After the sulfate attack, the sulfate ion reacts with calcium ion in pore solution. The leaching of solid-phase calcium supplements the reduction of calcium ions in pore solution until the solid-phase aluminate is completely dissolved [28], which leads to the decrease of the solid-phase calcium. It can be seen from Figure 13b, the solid-phase calcium in concrete less than 4.5 mm away from the ingress surface is completely consumed on the 180th day of sulfate attack, while the solid-phase calcium within the depth of 12.5 mm is completely consumed on the 360th day of sulfate attack. With the increase of sulfate ion ingress depth, the concentration of sulfate ion decreases, and the consumption of calcium ions in the pore solution decrease, resulting in the decrease of the consumption of solid-phase calcium in concrete. 

### 5.3. Influence of Solid-Phase Aluminate Dissolution

**Figure 14:**
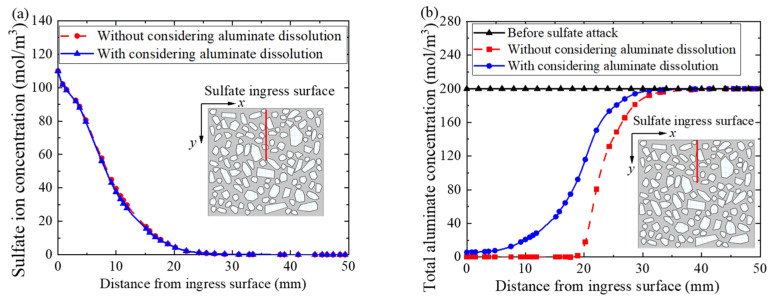
Effect of aluminate reaction and dissolution on sulfate attack. (**a**) Sulfate ion concentration distribution in concrete pore solution on the 360th day of sulfate attack, and (**b**) total aluminate concentration distribution in concrete on the 360th day of sulfate, respectively.

Figure 14a shows the concentrations of sulfate ion in concrete pore solution with and without considering the dissolution of solid-phase aluminate. Surprisingly, the concentrations of sulfate ion are almost the same in both cases, indicating that the dissolution of solid-phase aluminate has little influence on the distribution of sulfate ion concentration. The main reason could be attributed to the sulfate ion reacting with calcium ion to form gypsum, which belongs to the first-order chemical reaction. While the chemical reaction between gypsum and aluminate belongs to the second-order chemical reaction. Therefore, the dissolution of the solid-phase aluminate has little effect on the sulfate ion concentration distribution.

Additionally, before sulfate attack, the total aluminate concentration in concrete is uniformly distributed (herein 200 mol/m^3^), as shown by the black triangle solid line in Figure 14b. Without accounting for the dissolution of solid-phase aluminate, the initial concentration of aluminate in pore solution is the total concentration of aluminate in concrete. On the 360th day of sulfate attack, due to the chemical reaction, the aluminate in concrete is completely consumed in the area within 20 mm ingress depth, as shown by the red square dotted curve in Figure 14b. With the increase of sulfate ion ingress depth, the concentration of sulfate ion decreases, and the amount of gypsum also decreases, resulting in the decrease of aluminate consumption. However, when considering the dissolution of solid-phase aluminate, the initial concentration of aluminate in pore solution is lower than the total concentration of aluminate in concrete. Only the aluminate in the pore solution reacts with the gypsum generated by the chemical reaction between sulfate ion and calcium ion [28]. Therefore, the total aluminate concentration in concrete with considering dissolution of solid-phase aluminate (blue point solid curve in Figure 14b) is higher than that without considering the dissolution of solid-phase aluminate (red square dotted curve).

**Figure 15:**
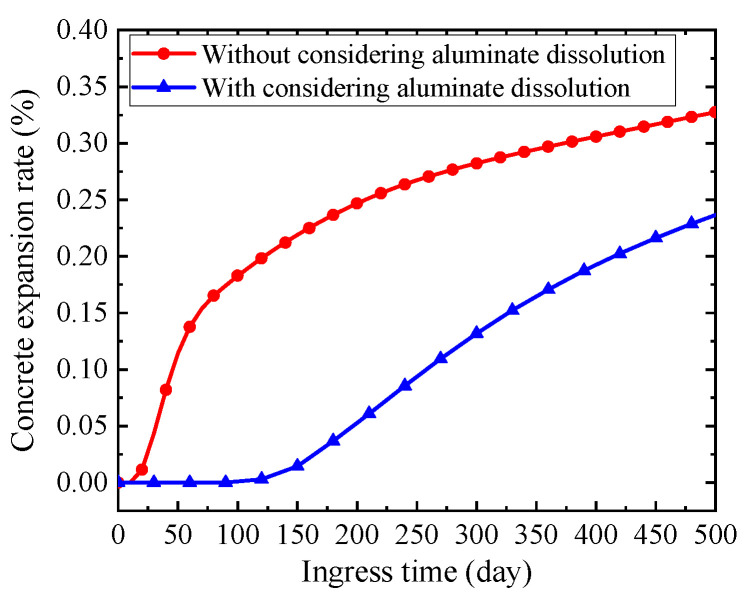
The concrete expansion rate with sulfate ingress time.

Moreover, the expansion rate of concrete with or without considering the dissolution of solid-phase aluminate is also demonstrated in Figure 15. The expansion rate of concrete without considering the dissolution of solid-phase aluminate increases rapidly in the initial stage of sulfate ingress (i.e., ingress time less than 150 days), and then increases smoothly in the later stage of sulfate ingress (i.e., ingress time more than 150 days), as the red point solid curve in Figure 15 shows. It can be explained that the initial total concentration of aluminate in pore solution is the maximum, and thus a large amount of ettringite is produced in the initial stage of sulfate ingress, resulting in the rapid expansion of concrete. While in the later stage of sulfate ingress, aluminate in pore solution is almost consumed and the ettringite is rarely formed, so that the expansion rate of concrete tends to be stable.

However, when considering the dissolution of solid-phase aluminate, the concrete expansion rate is almost zero in the initial stage of sulfate ingress (i.e., ingress time less than 150 days), and increases gradually in the later stage of sulfate ingress (i.e., ingress time more than 150 days), as the blue square solid curve in Figure 15 shows. It can be explained that the initial concentration of aluminate in pore solution and the concentration of sulfate ion are very low, so that there is nearly no ettringite in concrete and the expansion rate is nearly zero in the initial stage of sulfate ingress (i.e., ingress time less than 150 days). With the increase of sulfate ingress time (i.e., ingress time more than 150 days), the concentration of sulfate ion in concrete increases gradually and the consumed aluminate in pore solution is supplemented by the dissolution of solid-phase aluminate. Therefore, the ettringite produced gradually increases, and the expansion rate of concrete gradually increases. In addition, considering the dissolution of solid-phase aluminate obviously makes the expansion rate of concrete lower.

### 5.4. Influence of Boundary Sulfate Ion Concentration

**Figure 16:**
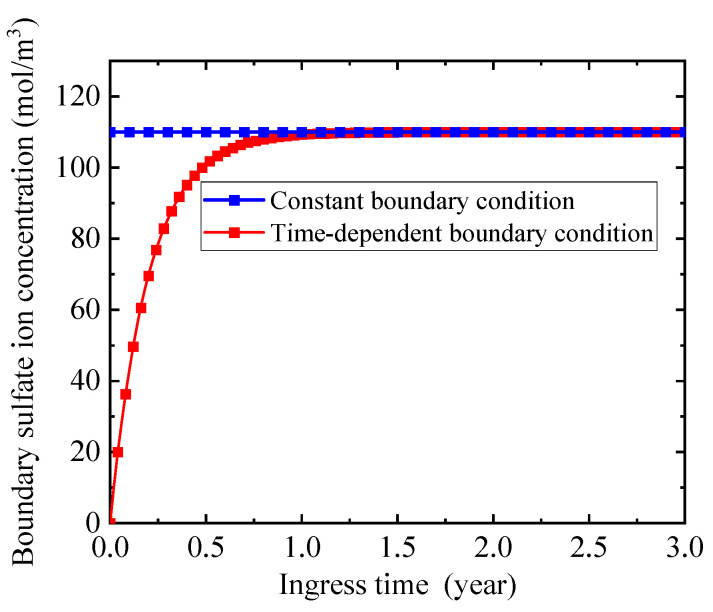
Sulfate ion concentration on the ingress surface.

**Figure 17:**
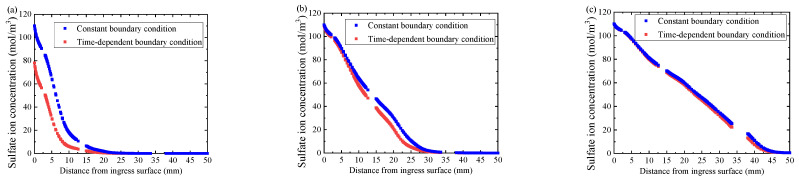
Sulfate ion concentration distribution in the pore solution on different ingress time. (**a**) 180th day, (**b**) 720th day, and (**c**) 1800th day, respectively.

**Figure 18:**
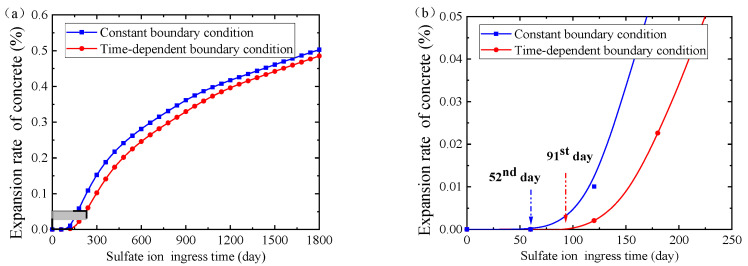
(**a**) History curves of concrete expansion rate along with sulfate ingress time, and (**b**) a local enlargement.

To investigate the influence of time-dependent boundary sulfate ion concentration on sulfate attack, the processes of sulfate attack in concrete with constant boundary condition and time-dependent boundary condition are numerically simulated for comparison, as shown in Figure 16, Figure 17 and Figure 18. In Figure 16, the constant boundary condition is shown by the blue square solid line, while the time-dependent boundary condition is plotted by the red square solid curve. The maximum sulfate ion concentration is selected as 110 mol/m^3^ [11] in the absence of special instructions. The time-dependent boundary condition is more consistent with the reality. The initial sulfate ion concentration on the concrete surface is far lower than that in the external environment of concrete at the initial stage of sulfate attack. With the increase of ingress time, it gradually increases and finally reaches the maximum value [71].

Figure 17a depicts the results of 180 days, the sulfate ions concentration in concrete with time-dependent boundary condition is obvious lower than that with constant boundary condition in the early stage of sulfate attack. Therefore, for short-term sulfate attacks, ignoring the time-dependent characteristics of boundary sulfate ion concentration will overestimate the diffusion performance of sulfate ions. However, as shown in Figure 17b,c, with the increase of sulfate ingress time, the difference of sulfate ion concentration of concrete with constant boundary condition and time-dependent boundary condition becomes smaller and smaller. Especially, the two sulfate concentration curves are almost the same after 1800 days of sulfate attack. This indicates that the time-varying characteristic of boundary sulfate ion concentration has little effect on the long-term sulfate attack.

Furthermore, the expansion rates of concrete with different boundary conditions have also been investigated as well. The expansion rate of concrete with constant boundary condition is higher than that with time-dependent boundary condition, but their difference gradually decreases with the increase of ingress time, as depicted in Figure 18a. Specifically, the difference of expansion rates is arising from the constant boundary or monotonic time-dependent boundary, which directly affects the sulfate ion concentration in concrete at the same ingress time and thus affects the amount of gypsum and ettringite generated. Consequently, the instant of expansion strain generation of concrete with constant boundary condition (i.e., 52nd day) is earlier than that of concrete with time-dependent boundary condition (i.e., 91st day), as shown in Figure 18b.

It is worth mentioning that the proposed numerical model of sulfate attack on concrete is applicable to ordinary concrete. Therefore, the numerical model may not be suitable for concrete with other types of concrete. This is because the mineral composition, pore structure, and mechanical properties of different types of concrete are different, which leads to the inconsistency of sulfate ion diffusion coefficient and chemical reaction caused by sulfate attack. Our findings reveal previously ignored fundamental aspects of the sulfate attack mechanism and provide insights for the durability prediction of RC structures. In the further study, relevant experimental research should be carried out to further verify and optimize the proposed model. Besides, the coupling effect of chloride and sulfate attack concrete.

## 6. Conclusions

In the present paper, a comprehensive multiphase mesoscopic model is proposed to fully reveal the chemical reaction-diffusion-mechanical mechanism of concrete under sulfate attack. Based on a systematic study, the following conclusions can be drawn:

(1) With the increase of sulfate ingress time, the calcium ion and the solid-phase calcium near the sulfate ingress surface are completely consumed, which promote the diffusion of sulfate ion.
(2) The dissolution of solid-phase aluminate has little influence on the distribution of sulfate ion concentration. However, the concrete expansion rate is overestimated if the dissolution of solid-phase aluminate is not modeled in the simulation.
(3) For short-term material performance assessment, the sulfate attack ability and the concrete expansion rate are overestimated if the time-dependent boundary of sulfate concentration is not taken into consideration.
(4) The sulfate ion in the mortar zone tends to diffuse into the ITZ, rather than directly diffuses in the mortar zone, indicating that ITZ is a fast channel for sulfate ion diffusion.

## Figures and Tables

**Table 1:** Main parameters in the proposed sulfate attack model.

Parameters	Value	References
Diffusion coefficient of sulfate ion, $D_{SO_{4}^{2-}}$	2.7 × 10^−11^ [m^2^/s]	[5]
Chemical reaction rate constant in Equation (1), $k_{1}$	3.05 × 10^−8^ [mol/m^3^ s]	[61]
Calcium ion concentration in the saturated liquid phase, $C_{Ca^{2+},sat}$	22 [mol/m^3^]	[35]
Diffusion coefficient of calcium ion, $D_{Ca^{2+}}$	2.7 × 10^−11^ [m^2^/s]	[5]
Chemical reaction rate constant in Equation (9), $k_{2}$	1.22 × 10^−9^ [mol/m^3^ s]	[61]
Diffusion coefficient of aluminate, $D_{CA}$	2.7 × 10^−11^ [m^2^/s]	*
Aluminate concentration in the saturated liquid phase, $C_{CA,sat}$	22 [mol/m^3^]	*
Model parameters in Equation (12), *k*	0.16 [--]	[16]
Model parameters in Equation (12), *m*	2.3 [--]	[16]
Volume fraction of the initial porosity being filled before the expansion0. in Equation (13)	0.05~0.4 [--]	[40]
Volume change rate of aluminate consumed per unit concentration, $v_{CA}$	1 × 10^−4^~1 × 10^−3^ [m^3^/mol]	[73]
Volume change rate of leaching unit calcium ions, $v_{CH}$	3.3 × 10^−5^ [m^3^/mol]	[61]
Threshold strain at which the microcracks start forming, $\varepsilon_{th}$	4 × 10^−5^ [--]	[16]

* Denotes an assumed value due to the lack of relevant experimental data. [--] represents dimensionless.

**Table 2:** Parameters obtained from the experimental data of Xie et al. [11].

*C_s,max_* [mol/m^3^]	*C_CA0_* [mol/m^3^]	*C_CH0_* [mol/m^3^]	*C_CSH0_* [mol/m^3^]	$v_{CA}\;[\mathrm{mol}/m^{3}]$	***q***	***f***
110	149.6	1445	4842	$4.2\times 10^{-4}$	3	0.23

**Table 3:** Parameters obtained from the experimental data of Rozière et al. [75].

*C_s,max_* [mol/m^3^]	*C_CA0_* [mol/m^3^]	*C_CH0_* [mol/m^3^]	*C_CSH0_* [mol/m^3^]	*a*_s_ [mol/m^3^]	*q*	*f*
201	141.3	1902	1807	$4.2\times 10^{-4}$	3	0.23

## Data Availability

The data used to support the findings of this study are available from the authors upon request.
